# Sequential Immunization with Vaccines Based on SARS-CoV-2 Virus-like Particles Induces Broadly Neutralizing Antibodies

**DOI:** 10.3390/vaccines12080927

**Published:** 2024-08-19

**Authors:** Youjun Mi, Kun Xu, Wenting Wang, Weize Kong, Xiaonan Xu, Xifeng Rong, Jiying Tan

**Affiliations:** 1 Department of Pathophysiology, School of BasicMedical Sciences, Lanzhou University, Lanzhou 730000, China; miyj@lzu.edu.cn; 2 State Key Laboratory for Animal Disease Control and Prevention, Lanzhou Veterinary Research Institute, Chinese Academy of Agricultural Sciences, Lanzhou 730046, China; wangwt2021@lzu.edu.cn (W.W.); kongwz19@lzu.edu.cn (W.K.); 220220926350@lzu.edu.cn (X.X.); rongxf2023@lzu.edu.cn (X.R.); 3 Gansu Provincial Key Laboratory of Evidence Based Medicine and Clinical Translation & Department of Immunology, School of Basic Medicine Sciences, Lanzhou University, Lanzhou 730000, China; tjykdxxukun@163.com; 4 People’s Hospital of Qianxinan Prefecture, Xingyi 562400, China

**Keywords:** virus-like particle (VLP), SARS-CoV-2 variant, prime-boost, sequential immunization, broadly neutralizing antibody

## Abstract

Although many people have been vaccinated against COVID-19, infections with SARS-CoV-2 seem hard to avoid. There is a need to develop more effective vaccines and immunization strategies against emerging variants of infectious diseases. To understand whether different immunization strategies using variants sequence-based virus-like particles (VLPs) vaccines could offer superior immunity against future SARS-CoV-2 variants, our team constructed VLPs for the original Wuhan-Hu-1 strain (prototype), Delta (δ) variant, and Omicron (ο) variant of SARS-CoV-2, using baculovirus-insect expression system. Then we used these VLPs to assess the immune responses induced by homologous prime-boost, heterologous prime-boost, and sequential immunizations strategies in a mouse model. Our results showed that the pro+δ+ο sequential strategies elicited better neutralizing antibody responses. These sequential strategies also take advantage of inducing CD4^+^ T and CD8^+^ T lymphocytes proliferation and tendency to cytokine of Th1. Currently, our data suggest that sequential immunization with VLPs of encoding spike protein derived from SARS-CoV-2 variants of concern may be a potential vaccine strategy against emerging diseases, such as “Disease X”.

## 1. Introduction

Following the COVID-19 pandemic in 2019, multiple new variants of SARS-CoV-2 have emerged, with some increasing the virus’s transmissibility. In response to this situation, several types of vaccines have been developed to prevent from COVID-19 [1,2,3]. According to data provided by WHO, as of early December 2023, there are 183 vaccine candidates in clinical development and 199 vaccine candidates in preclinical development [4]. These vaccine platforms include inactivated viruses, live attenuated viruses, protein subunits, viral vectors, RNA, DNA, virus-like particles (VLPs), and bacterial antigen-spore expression vectors [5,6,7,8,9]. The COVID-19 vaccine development strategy has primarily targeted the viral spike (S) protein of the Wuhan-Hu-1 strain (referred to as protype). All approved or authorized COVID-19 vaccines have been highly effective in reducing both COVID-19 morbidity and mortality [5]. Therefore, vaccinating the majority of the world’s population remains the most crucial means of combating the COVID-19 pandemic and reducing disease mortality.

Breakthrough infections have been common despite many people being vaccinated against COVID-19 [10,11,12]. On the one hand, some studies have reported that the antibody levels declined over time after vaccination [13,14,15]. On the other hand, the emergence of SARS-CoV-2 variants may reduce the efficacy of vaccines [16]. These were mutations of SARS-CoV-2. The Delta(δ) and Omicron(ο) variants are the key variants of concern (VOC) due to their significant mutations, but the neutralizing antibodies showed weak against the spike protein of these variants [17]. Therefore, there is a need to develop more effective vaccines and immunization strategies against new variants.

For some infectious diseases, achieving durable protective immunity usually requires the administration of two or more doses of the vaccine [18]. Prime-boost strategies may enhance waning immunity and extend the breadth of protection against SARS-CoV-2 variants [19,20,21]. Therefore, prime-boost COVID-19 vaccination regimens have been recommended in more and more countries [22].

VLPs are self-assembled viral protein complexes that mimic the morphological structure of virions. They lack the viral genome and are therefore noninfectious [23]. VLPs can display antigens on their surface and induce potent cellular and humoral immune responses [24]. VLP vaccines are considered safer than traditional inactivated and attenuated vaccines and are generally more immunogenic than subunit and recombinant protein vaccines due to their mimicry of the virus’s size and appearance, which effectively triggers both innate and adaptive immune systems [24]. mRNA vaccines require only the synthesis of antigenic RNA and are therefore fast to produce [25,26]. mRNA vaccines can be directly modified from the original sequence, allowing for timely updates in response to newly emerging variants [25,26]. mRNA vaccines are capable of eliciting both humoral and cellular immune responses [25,26]. This revolutionary technology is particularly suitable for emerging infectious diseases [25,26]. However, mRNA vaccines typically require ultra-low temperature cold-chain storage and transport to maintain the stability of the mRNA. mRNA vaccines may cause complications, especially myocarditis [27,28]. mRNA vaccine technology is relatively novel, but its long-term safety and efficacy data need more and long-term clinical trial [25,26]. In contrast, VLP vaccines are highly stable and generally do not require ultra-low temperature storage [7]. Additionally, VLP vaccines do not contain nucleic acid components and do not rely on lipid nanoparticles (LNPs) as a delivery system, thus avoiding potential side effects [7]. The safety and efficacy of VLP vaccines have been validated in multiple vaccines, such as the human papillomavirus (HPV) vaccine and the hepatitis B vaccine [8]. VLPs have shown great promise as a safe and effective vaccine platform [23]. There are 26 COVID-19 vaccines in clinical development based on the VLP platform according to the WHO landscape [4].

In our team’s previous studies, SARS-CoV-2 VLPs for the original Wuhan-Hu-1 strain (protype) have been successfully generated using a baculovirus-insect expression system, and cytological studies have shown that SARS-CoV-2 VLPs could induce dendritic cell maturation and modulate T cell immunity [29,30]. With Delta and Omicron becoming dominant in later stages of the COVID-19 pandemic, most vaccine efforts have focused on these variants. To address the potential emergence of virus variants, our group has developed a VLP platform based on various SARS-CoV-2 variants. Depending on the platform, it is possible to rapidly prepare VLPs vaccines against any SARS-CoV-2 variants even “disease X” for possible future virus epidemics.

This study further explores the potential of SARS-CoV-2 VLPs as vaccines by generating VLPs derived from these variants and assessing their immunogenicity using different immunization strategies.

## 2. Methods

### 2.1. Preparation of SARS-CoV-2 VLPs

SARS-CoV-2 VLPs were prepared according to our team’s previously reported method [29]. In brief, a pFastBac EMS triplex expression vector was constructed to generate SARS-CoV-2 VLPs based on any of the variants. The S genes from the prototype strain (Wuhan-Hu-1, GenBank accession No. MN908947.3), Delta (B.1.617.2, GenBank accession No. OR939692.1), and Omicron (B.1.1.529, GenBank accession No. OR939741.1) of SARS-CoV-2 were respectively cloned into the pFastBac EMS triplex expression vector in order to product prototype VLPs (pro-VLPs), Delta VLPs (δ-VLPs) and Omicron VLPs (ο-VLPs). Recombinant baculoviruses were then obtained by transfecting ExpiSf9™ insect cells with triplex expression vectors, respectively. The VLPs were generated by infecting insect cells with the recombinant baculovirus and were subsequently purified through sucrose density gradient centrifugation.

Purified VLPs preparations were adsorbed onto 200 mesh carbon-coated copper grids for 3 min, which were then incubated with rabbit anti-spike polyclonal antibody (40591-T62, SinoBiological, Beijing, China) for 1 h at room temperature. After washing the grids three times, the colloidal gold conjugated goat anti-mouse IgG (10 nm) (BOSTER, Wuhan, China) was incubated on the grid for another hour. Liquid was removed and the grids were negatively stained with 1% phosphotungstic acid. The morphology and characteristics of the VLPs were analyzed using a Talos F200C transmission electron microscope (TEM) (FEI, Brno, Czech Republic).

### 2.2. Mouse Immunization

**Figure 1:**
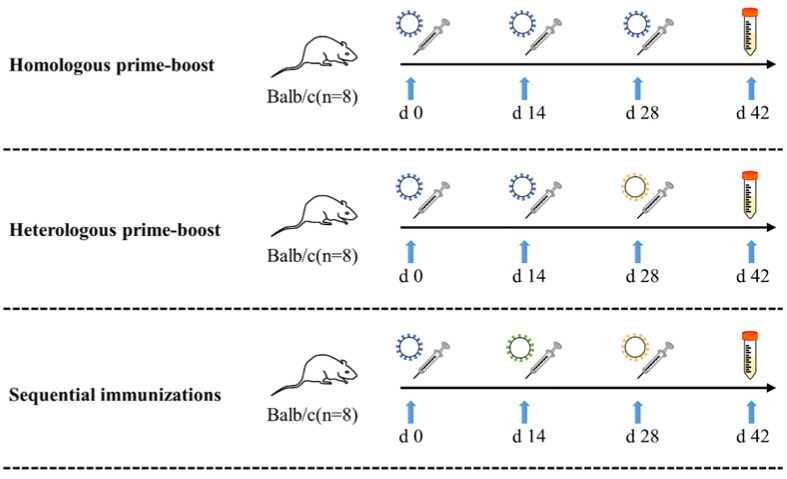
Immunization schedule of homologous prime-boost, heterologous prime-boost, and sequential immunizations strategies. Mice were initially immunized at 0 days, booster at 2-week intervals. The TEN buffer solution (TBS) was used as a control group. Detailed groups were shown in Table 1. Two weeks after final immunization (Day 42), all mice were sacrificed for test.

Three vaccination strategies with various VLPs, including homologous prime-boost, heterologous prime-boost, and sequential immunizations strategies, were designed in this study. Female Balb/c mice (n = 8) aged 6–8 weeks were immunized intramuscularly at Days 0, 14, and 21 (Figure 1). Mice were initially immunized with pro VLPs, boost immunization with different VLPs, including two booster doses pro-VLPs (homologous prime-boost, pro + pro + pro group), a booster dose of pro-VLPs and a booster dose of ο-VLPs (heterologous prime-boost, pro + pro + ο group), a booster dose of δ-VLPs and a booster dose of ο-VLPs (sequential immunizations, pro + δ + ο group). Taking into account the effect of the immunizing dose, each group was further subdivided into 5 μg, 10 uμg, and 50 μg dose subgroups. As a mock-vaccinated control group, another 8 mice were injected with three doses of TEN buffer solution (TBS, 200 μL/does). Table 1 lists the vaccination methods, immunogen dosages, and more details for each group. All murine experiments were approved by the Institutional Animal Care and Use Committee of Lanzhou University and were conducted in accordance with the guidelines of the Council on Animal Care and Use (jcyxy20200402).

### 2.3. The Evaluation of Antibody Responses in Sera

To evaluate the VLPs-induced antibody response, blood samples were collected from anesthetized mice via retroorbital vein bleeding two weeks after the last immunization. The samples were incubated at 37 °C for 1 h and then centrifuged at 2500 g for 15 min to recover the serum. These serums were used to determine the antibody response produced after the immunization. The IgG antibody responses were evaluated by enzyme linked immunosorbent assay (ELISA). Our study showed the S protein of protype(pro), Delta(δ), and Omicron(o) exhibition surface on those VLPs [29]. So, we utilized those VLPs to mimic S spike variants of SARS-CoV-2 for biological safety. 96-well plates were coated with 100 μL/well of the pro-VLPs, δ-VLPs and ο-VLPs at a concentration of 5 μg/mL, respectively, at 4 °C overnight, then blocked with 100 µL of 5% bovine serum albumin diluted in PBS at 37 °C for 1 h. Subsequently, sera samples were serially diluted in PBS, and 100 μL of serum dilution was added into each well of the plate in duplicate. The plate was incubated at 37 °C for 1 h and washed with PBST. Finally, the plate was incubated with goat anti-mouse IgG HPR secondary antibody diluted 1:10,000 in PBS at 37 °C for 1 h, and substrate tetramethylbenzidine peroxidase was used to develop. The absorbance was measured at 450 nm using an ELISA reader. Antibody levels were represented as the reciprocal of the geometric mean titer (GMT) based on the final serum dilution that had an optical density (OD) twice that of the control (normal) serum at the same dilution.

### 2.4. Surrogate Neutralization Assay

SARS-CoV-2 neutralizing antibodies were detected using SARS-CoV-2 neutralizing antibodies ELISA detection kits (EKnCov001, EKnCov002 and EKnCov003, Frdbio, Wuhan, China). This assay is a protein-based ELISA detection method designed to mimic the SARS-CoV-2-host interaction. This interaction between streptavidin-horseradish peroxidase (HRP)-conjugated SARS-CoV-2 spike protein receptor binding domain (RBD) and human angiotensin-converting enzyme 2 (ACE2) receptors can be blocked by neutralizing antibodies. The results of the NA test are presented as a percentage of inhibition. According to the manufacturer’s instructions, the cut-off value for antibody detection is recommended at 20% inhibition. In this study, we evaluated neutralizing antibodies against the SARS-CoV-2 wild-type strain, Delta variant, and Omicron variant following different vaccination strategies.

### 2.5. Immunophenotyping of Mouse Splenocytes

Spleens were aseptically removed from immunized mice from each group and gently homogenized in 5 mL Mouse 1× Lymphocyte Separation Medium (Dakewe Biotech, Shenzhen, China). The suspension was immediately transferred to a 15 mL centrifuge tube. 1 mL 1640 RPMI gently layered on top of the cell suspension, followed by centrifugation at 800× *g* for 30 min at 4 °C. The lymphocytes were harvested and resuspended in 10 mL of RPMI 1640. Then, the cell suspension centrifuged at 250× *g* for 10 min and the white cell pellet resuspended in 1 mL of chilled PBS.

For flow cytometry analysis, approximately 5 × 10^6^ cells were counted and transferred into 1.5 mL tubes. The cells were centrifuged and resuspended in a staining buffer consisting of 2% bovine serum albumin in 0.1 M PBS. FITC-conjugated anti-mouse CD4 antibody and PerCP-conjugated anti-mouse CD8 antibody (both from Invitrogen, Carlsbad, CA, USA) were added to the cells and incubated at 4 °C for 30 min. The stained cells were washed and resuspended in 450 μL PBS. Data were collected using a NovoCyte flow cytometer (Agilent Technologies, Santa Clara, CA, USA) and analyzed using NovoExpress analysis software version 1.5.6 (Agilent Technologies, Santa Clara, CA, USA).

### 2.6. Cytokine Analysis of Mouse Splenocytes

Spleens were collected and gently homogenized to generate single-cell splenocytes. To detect cytokines, splenocytes (3 × 10^6^ cells/well) were added to 24-well culture plates and stimulated with 5 μg VLPs at 37 °C with 5% CO_2_ for 3 days, with medium alone serving as the control. Culture supernatants were collected at 72 h for determination of IFN-γ and IL-4 levels. Secreted IFN-γ and IL-4 were quantified using the mouse IFN-γ (88-7314-22, Invitrogen, Carlsbad, CA, USA) and IL-4 (88-7044-22, Invitrogen, Carlsbad, CA, USA) uncoated Elisa kits according to the manufacturer’s instructions.

### 2.7. Hematoxylin and Eosin (H&E) Staining

For lung histopathology, all mice were euthanized and subjected to routine necropsy on days 14 post-immunization. Lung samples were harvested, fixed in a 10% buffered formalin solution, and were subsequently embedded in paraffin. The tissues were sectioned with a slicer and stained with hematoxylin and eosin (H&E). Histopathological observations were performed by a microscope.

### 2.8. Statistical Analysis

Statistical analyses were performed with GraphPad Prism software V9.0.0 (Dotmatics, Boston, MA, USA) and SPSS version 21.0 (IBM, New York, NY, USA). Analysis of variance (ANOVA) with a Tukey-Kramer post hoc test was performed for the comparison of frequencies of T cells between groups. For the comparison of VLP-specific IgG and neutralizing antibody levels, as well as cytokine secretion from immunized mouse splenocytes, we used the Kruskal-Wallis test with Dunn’s multiple-comparison test. A *p* value < 0.05 was considered statistically significant.

## 3. Results

### 3.1. Generation of SARS-CoV-2 VLPs

**Figure 2:**
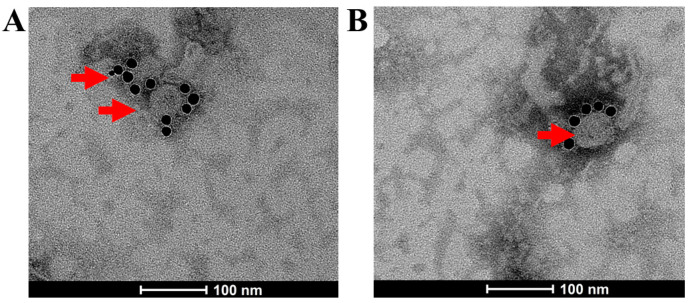
Morphology of SARS-CoV-2 VLPs as shown in transmission electron microscope. The arrows denote VLPs. (**A**) Electron microscopy reveals δ-VLPs specifically labeled with immunogold. (**B**) Electron microscopy reveals ο-VLPs specifically labeled with immunogold. Scale bar = 100 nm.

In our previous work, we successfully generated VLPs base on SARS-CoV-2 Wuhan-Hu-1 strain(protype) by an insect expression system [29]. Based on this work, we aimed to develop a VLP platform for various SARS-CoV-2 variants. For the modular production of VLPs for SARS-CoV-2, the *S* genes from protype(pro), Delta(δ) variant, and Omicron(o) variant were individually inserted into pFastBac EMS triplex expression vector and SARS-CoV-2 VLPs based on variants were generated in insect cells using the Bac-to-Bac system. The VLPs were visualized by negative staining under a transmission electron microscope, with the S protein location indicated by immunogold labeling using an S protein-specific antibody (Figure 2). As shown in Figure 2A,B, the SARS-CoV-2 VLPs appeared as spherical particles; the VLPs size is between 49 nm and 97 nm.

### 3.2. VLPs Induced Antibody Responses

**Figure 3:**
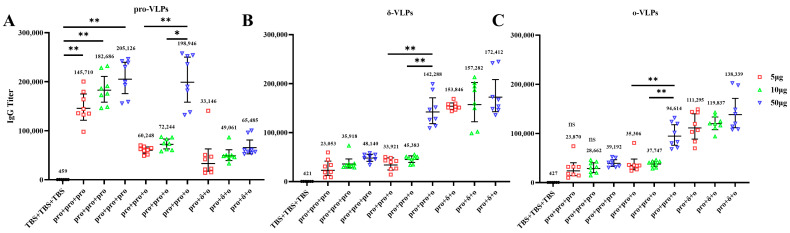
S protein on VLPs specific pan-IgG endpoint titers were measured at week 2 after the final immunization. Pan-IgG immune responses were measured using ELISA. (**A**–**C**) 96-well plates were coated with pro-VLPs, δ-VLPs, ο-VLPs respectively. Subsequently, the sera samples were diluted in series in PBS and added into each well of the different plate for test. GMT and 95% confidence interval (CI) are presented. The Kruskal-Wallis with Dunn’s multiple-comparison test was performed for comparison of VLPs-specific IgG between groups. * *p* < 0.05, ** *p* < 0.01.

To examine the induction of antibodies against different VLPs, sera from immunized mice were assayed for the presence of VLPs-specific total IgG. As shown in Figure 3, VLPs-matched endpoint IgG titers (geometric mean titer, GMT) were significantly increased in mice after the final immunization at week 2. Higher IgG levels against pro-VLPs and low IgG response against δ-VLPs or ο-VLPs were detected in pro + pro + pro group (Figure 3). Compared to the 5 μg and 10 μg subgroups, the 50 μg subgroup of the pro + pro + ο group elicited higher IgG levels against different VLPs formulations (Figure 3A–C). In pro + δ + ο group, higher IgG levels against δ-VLPs or ο-VLPs and weak IgG response against pro-VLPs was detected (Figure 3B,C). Negative control mice receiving TBS showed no IgG antibodies against any VLPs. These results indicate that all prime-boosts regime with VLPs enhanced antibody responses.

**Figure 4:**
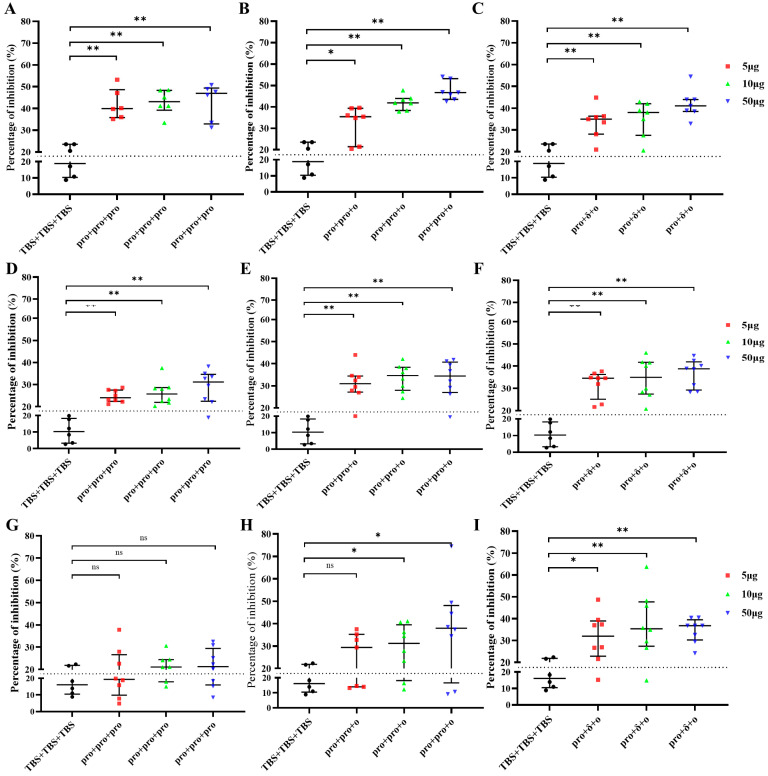
Neutralizing activity against the wild type, Delta variant, and Omicron variant measured by a surrogate ELISA neutralization assay. (**A**–**C**) different immunization strategies induced neutralizing activity against the RBD of wild strain. (**D**–**F**) different immunization strategies induced neutralizing activity against the RBD of Delta variant. (**G**–**I**) different immunization strategies induced neutralizing activity against the RBD of Omicron variant. Medians and interquartile ranges are presented. The Kruskal-Wallis with Dunn’s multiple-comparison test was performed for comparison of neutralizing activity between groups. Asterisks on the graph represent significant differences between immunized group and TBS + TBS + TBS control (* *p* < 0.05 and ** *p* < 0.01. ns, no significant differences).

To evaluate the neutralizing potential of antibodies induced by different immunization strategies, a surrogate neutralization assay was carried out using homologous or heterologous RBD proteins. The pro + pro + pro group elicited neutralizing activity against the RBD of Wuhan-Hu-1 (Figure 4A) and RBD of Delta (Figure 4D), but no neutralizing activity against the RBD of Omicron (Figure 4G). The pro + pro + ο group triggered neutralizing activity against the RBD of Wuhan-Hu-1 (Figure 4B) and RBD of Delta (Figure 4E). However, this group required a higher immunizing dose of VLPs (10 μg or 50 μg) to induce neutralizing activity against Omicron’s RBD (Figure 4H). The pro + δ+ο group induced neutralizing antibodies against all tested homologous or the heterologous RBD (Figure 4C,F,I).

### 3.3. Cellular Immune Responses Elicited by Different Immunization

**Figure 5:**
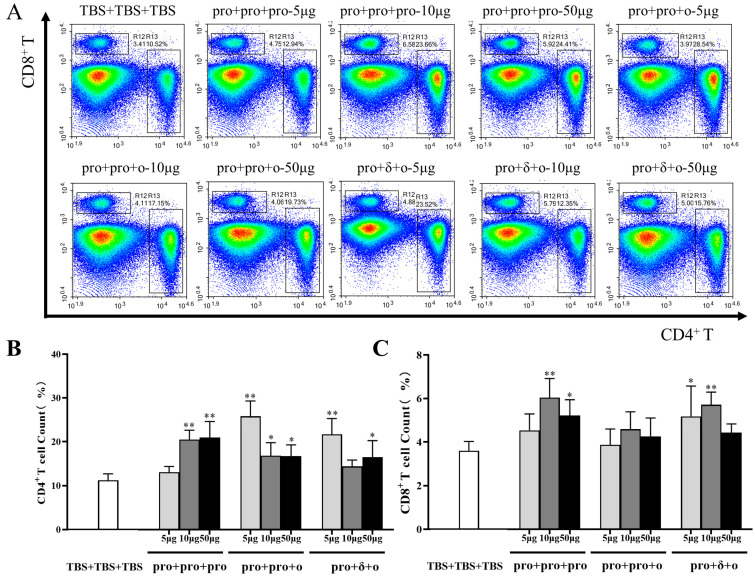
Immunophenotyping of mouse splenocytes. Representative flow cytometry plots for the cell sorting of CD4+ and CD8+ T cells from each group (**A**). The ratio of CD4+ (**B**) and CD8+ (**C**) T cells isolated from the mouse splenocytes. Data depict the mean for six mice. Error bars represent SD. Analysis of variance (ANOVA) with a Tukey-Kramer post hoc test was performed for comparison of frequencies of T cells between groups. Asterisks on the graph represent significant differences between immunized group and TBS + TBS + TBS control (* *p* < 0.05 and ** *p* < 0.01).

In addition to evaluating humoral immune responses, T cell frequencies were analyzed using flow cytometric. Except for the 5 μg pro + pro + pro group and 10 μg pro + δ + ο group, all the other groups exhibited a significant increase in CD4+T cell frequencies compared to the control group (Figure 5A,B). After boosting, CD8+T cell frequencies were increased in the 10 μg and 50 pro + pro + pro groups, as well as the 5 μg and 10 μg pro + pro + ο groups. No significant differences were observed between the pro + pro + ο groups and the control group (Figure 5A,C).

**Figure 6:**
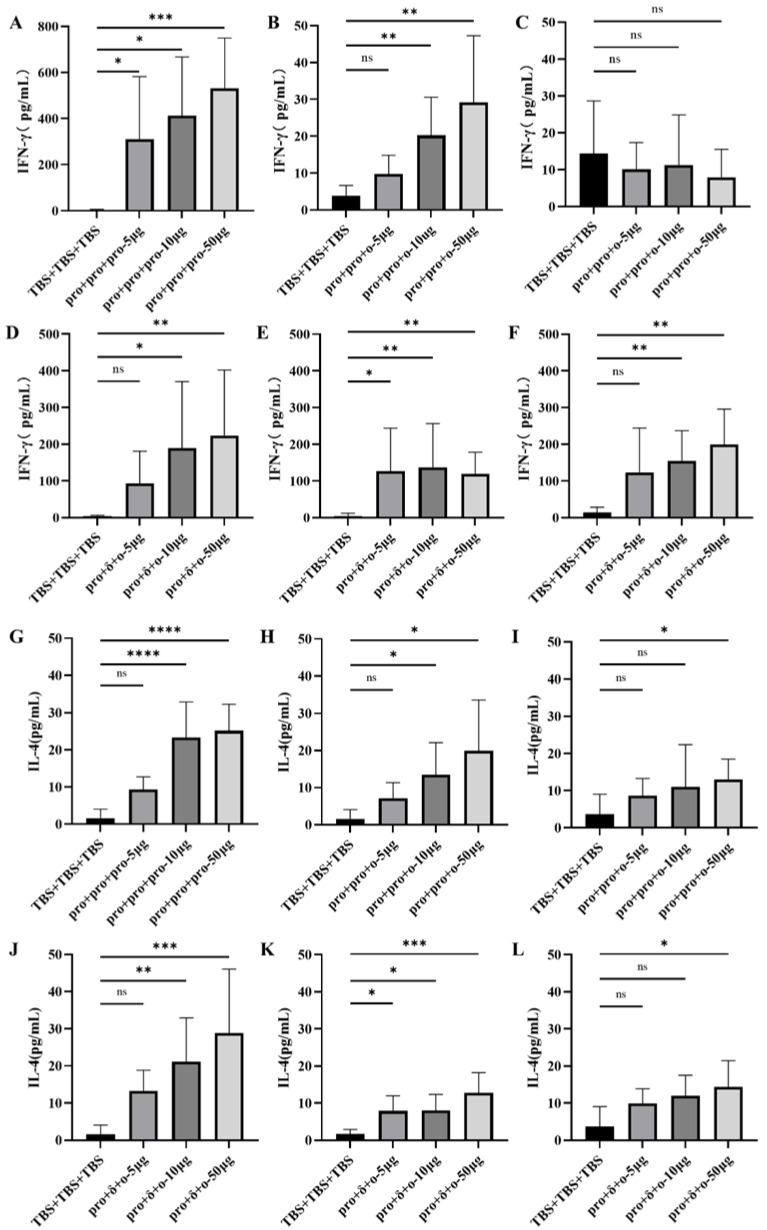
Cytokine secretion from immunized mouse splenocytes. Splenocytes were isolated from each group after the second booster immunization. Cells were seeded into 96-well cell culture plates and pro-VLPs (**A**,**B**,**D**,**G**,**H**,**J**), δ-VLPs (**E**,**K**), ο-VLPs (**C**,**F**,**I**,**L**) were added into cell culture medium, and IFN-γ and IL-4 were determined as described in Materials and Methods. Data represented the mean S.D. for six mice. The Kruskal-Wallis with Dunn’s multiple-comparison test was performed for comparison of cytokine secretion between groups. Asterisks on the graph represent significant differences between immunized group and TBS + TBS + TBS control (* *p* < 0.05, ** *p* < 0.01, *** *p* < 0.001; **** *p* < 0.0001. ns, no significant differences).

To further investigate the cellular immune responses, the Th1(IFN-γ) and Th2 (IL-4) cytokine levels were measured in the supernatants of cultured splenocytes from vaccinated mice. Splenocytes from the pro + pro + pro group, when stimulated with pro-VLPs, showed a significantly increased INF-γ level compared to other VLP-immunized groups or the control group (*p* < 0.01) (Figure 6A). The splenocytes of mice in the pro + pro + ο group stimulated with pro-VLPs or ο-VLPs elicited low levels of INF-γ (Figure 6B) and no INF-γ (Figure 6C) production, respectively. Except for the 5 μg pro + δ+ο group, the splenocytes of mice in the pro + δ + ο group stimulated with any VLPs can elicited significantly increased INF-γ level compared with the control group (Figure 6D–F).

The splenocytes of mice in all 10 μg and 50 group stimulated with pro-VLPs elicited significantly increased IL-4 levels (Figure 6G,H,J). The 50 μg pro + pro + ο group and the pro + δ + ο group stimulated with ο-VLPs can elicited significantly increased IL-4 levels (Figure 6I,L). pro + δ + ο group stimulated with δ-VLPs can elicited significantly increased IL-4 levels (Figure 6K).

### 3.4. Histopathologic Analysis

**Figure 7:**
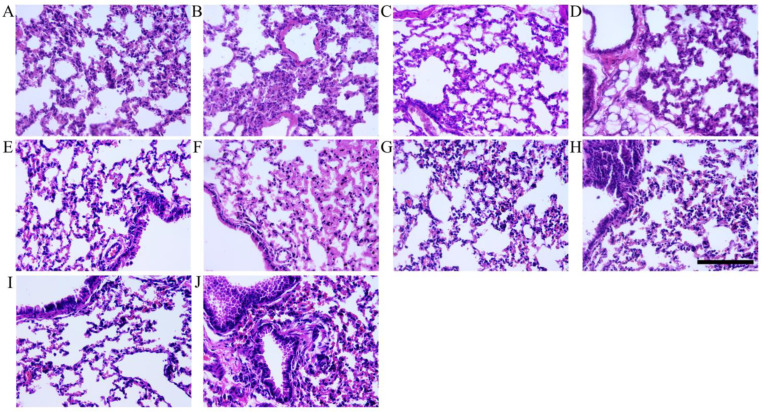
Lung histopathology of the control and immunized mice at 14 days after immunization. Mice were inoculated with the TBS (**A**), immunized with 5 μg (**B**), 10 μg (**C**), 50 μg (**D**), pro-VLPs using a homologous prime-boost strategy, vaccinated with 5 μg (**E**), 10 μg (**F**), 50 μg (**G**) pro-VLPs, and ο-VLPs in a heterologous prime-boost regimen, or inoculated with 5 μg (**H**), 10 μg (**I**), 50 μg (**J**) pro-VLPs, δ-VLPs, and ο-VLPs using a sequential immunizations approach. Scale bar: 50 μm.

In our previous study, golden gophers developed significant pulmonary edema following the intramuscular administration of high-dose injections of VLPs (unpublished). In order to assess whether different immunization strategies with VLPs induce similar immunopathology effects, we examined lung histological lesions in mice 14 days after vaccination. The lung tissues were sectioned with a slicer and stained with hematoxylin and eosin (H&E). Mice from all VLP-inoculated groups did not exhibit lung lesions compared to the control group (see Figure 7).

## 4. Discussion

Since the onset of the COVID-19 pandemic in 2019, multiple new variants of SARS-CoV-2 have emerged and rapidly spread across the globe [1]. Developing a highly effective vaccine is a practical approach for controlling the COVID-19 pandemic. Various platforms have been utilized in the development of COVID-19 vaccines such as mRNA, protein subunit vaccines, VLPs, etc. [3,16]. Producing VLP-based vaccines is more expensive and requires complex and precise procedures compared to other vaccines, and the isolation and purification of VLPs are also challenging [31]. However, as mentioned earlier, VLPs has unique advantages that make it an important platform for the development of COVID-19 vaccines as well. Up to early December 2023, there were 26 VLP-based COVID-19 vaccines in the preclinical phase and 7 in clinical development.

The S glycoprotein is the main target for COVID-19 VLP vaccine development. SARS-CoV-2 has consistently mutated throughout the pandemic, resulting in variants that differ significantly from the original virus [32,33]. Amino acid substitutions and deletions in the S protein may can enhance the virus’s ability to evade neutralizing antibodies, potentially reducing vaccine efficacy [33,34]. Therefore, we developed a platform to rapidly generate VLPs for various variants by replacing S genes from any variant. In this way, we build up a technical reserve for possible future virus epidemics.

Prime vaccination alone may not consistently activate the immune system, and the immunity provided by the initial dose can wane over time. Therefore, a prime-boost scheme is necessary for COVID-19 vaccinations, and administering a booster vaccine is essential to address newly emerged SARS-CoV-2 variants [35]. Since a prime-boost vaccination strategy is effective in enhancing both cellular and humoral immune responses, it has been widely employed in developing vaccines against infectious diseases such as HIV, HBV, HCV, malaria, and TB [36,37,38,39]. Homologous prime-boost strategy uses the same formulation for both the prime and boost phases, whereas the heterologous prime-boost regimen involves different formulations for the prime and boost administrations [36]. Numerous studies have shown that heterologous vaccination schedules are more effective than homologous immunization [21,33,36]. In this study, according to the population vaccination trends and the prevalence of variants, different prime-booster strategies were designed to evaluate the immunization efficacy of VLP vaccines derived from different SARS-CoV-2 variants. Our results demonstrated that the pro + pro + pro homologous prime-boost regimen could induce an IgG response against all VLPs. However, this regimen was not successful in generating a broadly neutralizing antibody responses against SARS-CoV-2 variants. For example, immune sera from mice vaccinated with prototype VLPs showed neutralizing activity against the RBD of autologous SARS-CoV-2 and the RBD of the Delta variant, but no neutralizing activity against the RBD of the Omicron variant (Figure 4G). Germain Simon et al. analyzed serum samples from healthcare workers who treated with a homologous prime-boost of BNT162b2 mRNA vaccine. After 6 months, the percentage of inhibition in the surrogate neutralization assay was 98.6% for the wild-type virus and 53.4% for the Omicron BA.1 variant [40]. According to Martin A Prusinkiewicz et al., while increasing the interval between vaccine doses can enhance vaccine immunogenicity, the median percentage of inhibition in the surrogate neutralization was 99% for the wild-type strain after three doses of BNT162b2 mRNA vaccine, compared to 61% for the Omicron BA.1 variant [41]. These results suggest that the SARS-CoV-2 Omicron variant escapes the pro + pro + pro strategy-induced antibody neutralization. Several studies also have confirmed a marked reduction in the neutralizing ability of vaccine-induced antibodies against Omicron [21,42]. Research by Jingwen Ai demonstrated that the Omicron variant is more susceptible to evading immunity induced by inactivated vaccines compared to the prototype strain and other VOCs [43]. According to Emma K. Accorsi et al., even after receipt of 3 doses of the COVID-19 mRNA vaccine, the estimated relative effectiveness against Omicron and Delta was 66.3% and 84.5%, respectively [44]. The multiple deletions and mutations in the S protein confer on the omicron variant a powerful ability to evade vaccine-induced humoral immune response [45]. The RBD mutations K417N, E484A, and Y505H in Omicron are likely to reduce the binding affinity of most antibodies to the S protein, suggesting that Omicron has a greater potential for vaccine breakthrough compared to other variants [46].

When mice were immunized with the pro + pro + ο heterologous prime-boost strategy, neutralizing responses against the Omicron RBD were observed (Figure 4). This immunization strategy also induced a neutralizing antibody response against Delta RBD (Figure 4E), suggesting that the breadth of pro + pro + ο-induced neutralizing antibodies was not compromised compared with pro + pro + pro immunization. Consistent with the findings reported by Baoling Ying, mice vaccinated with two doses of mRNA-1273 followed by a booster dose of mRNA-1273.529 (an Omicron-matched vaccine) exhibited increased neutralizing antibody titers and enhanced protection against Omicron BA.1 and BA.2 infections [47]. Many researchers have confirmed that heterologous prime-boost provides greater immunity than homologous prime-boost [48,49]. Notably, pro + δ + ο sequential vaccination, based on pro, Delta, and Omicron VLPs, exhibited superior ability in inducing neutralizing antibody responses against all tested RBDs (Figure 4). Sequential immunization is more effective at inducing neutralizing antibodies against the Omicron (BA.2) variant in mice compared to other immunization strategies [50].Ting Zhang et al. reported that sequential immunization with 2RBD-Fc, 2dRBD-Fc, and 2omRBD-Fc DNA vaccines based on different variants effectively induced neutralizing antibodies against each immunized variant, as well as RBD-specific T cell responses [51]. These findings suggest that a sequential immunization strategy may be necessary to achieve the required breadth of humoral responses. The extensive neutralizing antibody responses against SARS-CoV-2 variants observed with sequential immunizations could be attributed to the targeting of conserved spike protein epitopes and the introduction of new epitopes.

The ability to elicit neutralizing antibodies against the S proteins of SARS-CoV-2 is a major component of immune protection, but this alone is not sufficient to limit the spread of the virus [52]. Several studies have suggested that T cell responses play an important role in protecting against SARS-CoV-2 [53,54]. Therefore, we aimed to investigate cellular immune responses in detail after different prime-boost vaccination regimens. We analyzed CD4+ and CD8+ T cell subsets in the splenocyte to compare the effectiveness of various vaccine regimens. In this study, all groups inoculated with VLPs triggered a significant proliferation of CD4+ T cells compared to the control groups. At the same time, the level of CD8+ T cells was higher in the pro + pro + pro group and the pro + δ + ο group compared with the control group, except for the pro + pro + ο group. CD4+ T cells immune responses play an important role in suppressing initial SARS-CoV-2 infections and their correlation with reduced COVID-19 severity [55,56]. CD8+ T cells are key to virus clearance due to their ability to destroy virus-infected cells. Cytokines produced by T cells partly mediate the immune response after vaccination. In line with the changes in T cell subsets, the production of IFN-γ and IL-4 was lower when splenocytes were restimulated with ο-VLPs in the pro + pro + ο group. Above all, the results of both humoral and cellular responses imply that sequential immunization may be a potential strategy to enhance SARS-CoV-2-specific immune responses.

In conclusion, our study evaluated the humoral and cellular responses evoked by three vaccination strategies using VLPs vaccines in a mouse model. The sequential immunizations regimens demonstrated superior immune responses compared to other strategies in mice. These findings suggest that sequential vaccination may be an effective approach for enhancing immune protection against SARS-CoV-2 variants.

## Figures and Tables

**Table 1:** Vaccination strategies and dosage of immunogens.

Vaccination Strategy	Group	μg/Dose	First Dose	Second Dose	Third Dose	*n*
Control	TBS + TBS + TBS	-	TBS	TBS	TBS	8
Homologous prime-boost	pro + pro + pro	5	pro-VLPs	pro-VLPs	pro-VLPs	8
		10	pro-VLPs	pro-VLPs	pro-VLPs	8
		50	pro-VLPs	pro-VLPs	pro-VLPs	8
Heterologous prime-boost	pro + pro + ο	5	pro-VLPs	pro-VLPs	ο-VLPs	8
		10	pro-VLPs	pro-VLPs	ο-VLPs	8
		50	pro-VLPs	pro-VLPs	ο-VLPs	8
Sequential immunizations	pro + δ + ο	5	pro-VLPs	δ-VLPs	ο-VLPs	8
		10	pro-VLPs	δ-VLPs	ο-VLPs	8
		50	pro-VLPs	δ-VLPs	ο-VLPs	8

Abbreviation: TBS, TEN buffer solution. pro protype. δ, Delta. ο, Omicron.

## Data Availability

The data presented in this study are available on request from the corresponding author.
